# Death Receptors: New Opportunities in Cancer Therapy.

**Published:** 2017

**Authors:** V.M. Ukrainskaya, A.V. Stepanov, I.S. Glagoleva, V.D. Knorre, A.A. Jr. Belogurov, A.G. Gabibov

**Affiliations:** M.M. Shemyakin and Yu.A. Ovchinnikov Institute of Bioorganic Chemistry, Miklukho-Maklaya Str., 16 /10, Russian Academy of Sciences, Moscow, 117997, Russia; Institute of Fundamental Medicine and Biology, Kremlyovskaya Str., 18, Kazan Federal University, Kazan, 420008, Russia

**Keywords:** apoptosis, death receptors, DR4, DR5, TNF, tumor cells

## Abstract

This article offers a detailed review of the current approaches to anticancer therapy that target the death receptors of malignant cells. Here, we provide a comprehensive overview of the structure and function of death receptors and their ligands, describe the current and latest trends in the development of death receptor agonists, and perform their comparative analysis. In addition, we discuss the DR4 and DR5 agonistic antibodies that are being evaluated at various stages of clinical trials. Finally, we conclude by stating that death receptor agonists may be improved through increasing their stability, solubility, and elimination half-life, as well as by overcoming the resistance of tumor cells. What’s more, effective application of these antibodies requires a more detailed study of their use in combination with other anticancer agents.

## INTRODUCTION

The major current approaches to cancer therapy are based on a combination of chemotherapy and surgery. But, because of the lack of cancer specificity, they are often associated with a variety of severe side-effects and complications. For this reason, the design of highly specific drugs, such as monoclonal antibodies, for a targeted inhibition of cancer cells appears to be a very promising direction [1]. A better understanding of tumor biology and tumor immunology affords us the opportunity to use apoptosis as a target for the future development of selective anticancer agents. Apoptosis is a natural physiological process that controls the number of cells in tissues and plays a key role in the elimination of damaged, unwanted, and diseased cells. However, the malignant transformation of cells often disrupts apoptosis pathways [2]. It is noteworthy that our growing understanding of the mechanisms that regulate programmed cell death has led to the emergence of new agents capable of restarting apoptosis in malignant cells. A major proportion of current therapeutic agents capable of initiating apoptosis comprises low-molecular-weight compounds, the disadvantages of which are systemic complications [3].

A fundamentally different approach to anticancer therapy is the search for tumor necrosis factor receptor superfamily (TNFRSF) agonists. So-called death receptors containing a death domain comprise a separate group of the superfamily. These include the tumor necrosis factor receptor 1 (TNFR1), tumor necrosis factor receptor 6 (CD95, FasR, APO-1), death receptor 4 (DR4), death receptor 5 (DR5), etc. Receptors DR4 and DR5 are the most promising candidates for targeted therapy of tumor diseases, because their expression levels are significantly higher in cancer cells than in normal ones [4, 5]. Therefore, unlike chemotherapeutic agents, these receptors may potentially mediate selective killing of tumor cells.

In normal cells, the apoptotic mechanisms are regulated by anti-apoptotic proteins: for example, the cellular FLICE-like inhibitory protein (c-FLIP) suppresses caspase 8 activation, and Bcl-2 family proteins, forming part of a heterocomplex with caspases, and inhibit the apoptotic signal [6, 7].

## THE STRUCTURE OF THE DEATH RECEPTORS 4 AND 5

DR4 and DR5 are type I transmembrane proteins consisting of three (extracellular, transmembrane, and intracellular) domains. The last domain comprises a homologous cytoplasmic sequence of the death domain. Furthermore, DR5 can exist as two isoforms, DR5 (L) and DR5 (S): the short form lacks 29 amino acid residues between the cysteine sequences and the transmembrane region, but this does not affect the functional activity of the receptor [8].

DR4 and DR5 receptors are found in cells of various human tissues, including thymus, liver, leukocytes, activated T cells, and small intestine. They are also detected in some tumor lines, such as Jurkat [9], Ramos [10], HeLa [11], Colo205 [12], etc. Identity of the death and cysteine-rich domains of DR4 and DR5 is 64% and 66%, respectively [13].

**Fig. 1:**
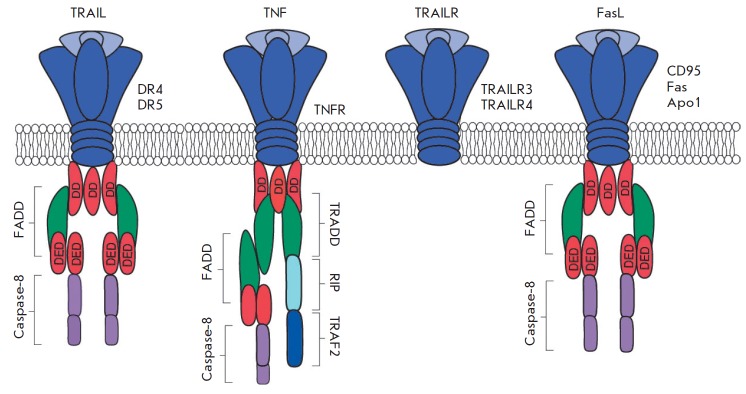
Structures of death receptors. Death receptors and their ligands include: receptors DR4 (TNFRSF10A, TRAIL-R1), DR5 (TNFRSF10B, TRAIL-R2, Apo2), DcR1 (TRAILR3), and DcR2 (TRAILR4) and their ligand, TRAIL; the tumor necrosis factor receptor (TNFR) and its ligand, the tumor necrosis factor (TNF); the Fas receptor (CD95, Apo1) and its ligand, FasL. Note: TRADD – the tumor necrosis factor receptor type 1-associated death domain protein; FADD – the Fas-associated death domain protein; DD – a death domain; DED – a death effector domain; RIP – a receptor-interacting protein.

The interaction between a receptor and a ligand (TRAIL/Apo2L) occurs first at the N-terminus of the extracellular domain, when the ligand binds to a first cysteine domain, the so-called pre-ligand assembly domain (PLAD) [14]. This sequence is not directly involved in receptor oligomerization, but it stabilizes a ligand relative to the receptor [15]. Previously, ligand trimerization was determined to occur in the presence of a Zn^+2^ ion [16] that non-covalently binds to the cysteine-rich domains of TRAIL. Stabilization of TRAIL is accompanied by a conformational change in the monomeric receptor, followed by translocation of the receptor into membrane lipid rafts and the formation of its active trimeric form [17]. Then, an adaptor protein associates with the receptor through the homotypic interaction between the adaptor’s death domain and the receptor’s death domain (DD-DD). Adaptor molecules include the Fas-associated DD (FADD) protein that interacts with a death domain of the Fas receptor and the TNFR1-associated DD (TRADD) protein that interacts with a death domain of the TNFR1 receptor [18]. TRADD and FADD also comprise additional protein interaction modules called death effector domains (DEDs) [19]. They can associate with procaspases 8/10 and the regulatory protein c-FLIP. The multiprotein complex formed between the death domain of the FADD receptor and caspases 8/10 is called the death-inducing signaling complex (DISC) [20] (*Fig. 1*). After the formation of DISC, the apoptotic signal is transmitted to initiator caspases.

## ACTIVATION OF APOPTOSIS

Apoptosis is a complex energy-consuming process involving a cascade of molecular transformations. To date, two, mitochondrial and receptor-mediated, apoptotic pathways are known.

After the DISC formation, the apoptotic signal is transmitted to initiator caspases. Caspases occur in the cell as inactive procaspases (32–56 kDa) that are monomers consisting of a N-terminal domain, large (17–21 kDa) and small (10–13 kDa) subunits, and short linking regions [21]. There are several theories of the caspase activation process. According to one of them, clustering of caspases at the DISC leads to their self-activation through autocatalytic processing. According to another theory, assembling of initiator caspases promotes their dimerization, which results in cleavage of the N-terminal pro-domain and linking regions in each monomer, with the large and small subunits forming heterodimers [22]. High local concentrations of initiator procaspases induce their binding to the FADD domain.

The substrate specificity of initiator caspases is limited by effector caspases and the pro-apoptotic Bid protein [23]. Activation of DISC-associated caspases 8/10 promotes subsequent activation of the effector caspases 3 and 7 exhibiting enzymatic activity. The effector caspase cleavage site is an Asp residue in a tetrapeptide motif [24, 25]. Activation of effector caspases triggers a variety of signaling pathways that control cell activity.

**Fig. 2:**
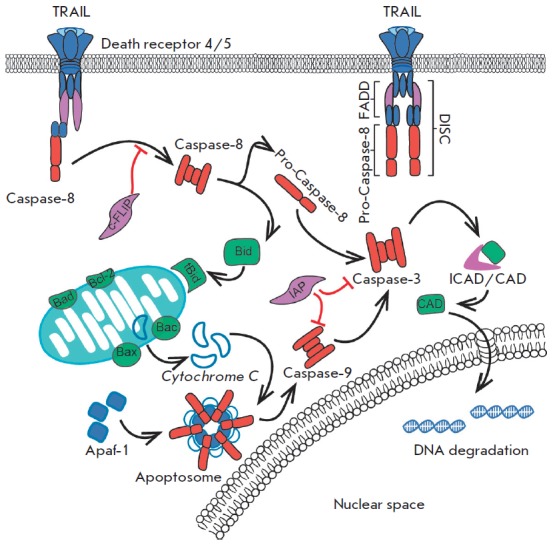
Apoptosis signal transduction pathways (receptor- mediated and mitochondrial pathways): receptor- ligand interaction leads to DISC formation, which induces factors that activate apoptosis (caspase 8, caspase 3, etc.). The release of cytochrome c leads to apoptosome formation and activation of caspase 9. Note: DISC – the death-inducing signaling complex; Bid, Bad, Bcl-2, and Bac – Bcl2 protein family; ICAD\CAD – caspase activated DNAse; Apaf-1 – the apoptotic protease activating factor 1; IAP and c-FLIP – apoptosis inhibitory proteins.

The mitochondrial apoptotic pathway is most often activated by intracellular factors in response to various signals: DNA damage, formation of reactive oxygen species, accumulation of misfolded proteins, etc. This process is regulated by the proteins of the Bcl-2 family. The family includes the Bid factor that is cleaved and activated by caspase 8 [26]. The activated form of Bid (tBid) causes permeabilization of the mitochondrial membrane, release of cytochrome *c*, and formation of the apoptosome that activates initiator caspase 9 [27]. This is a key moment in the development of intracellular apoptosis, which leads to the activation of effector caspases (*Fig. 2*).

Both the receptor-mediated and mitochondrial pathways lead to the activation of cytoplasmic DNA-degrading endonucleases and proteases that destroy intracellular proteins. Caspases 3, 6, and 7 directly cleave cytokeratin and the cell membrane, which leads to the morphological changes seen in any apoptotic cell [28].

## TRAIL

Like the tumor necrosis factor (TNF), TRAIL belongs to the tumor necrosis factor superfamily (TNFSF) and participates in the regulation of vital biological functions in vertebrates [29]. Being a ligand of DR4 and DR5, TRAIL comprises two antiparallel beta-pleated sheets that form a beta-sandwich [30]. Containing the only cysteine residue, TRAIL is capable of chelating zinc. Subunits interact with each other in a head-to-tail fashion to form a homotrimer resembling a truncated pyramid [31]. TRAIL also contains a significant number of aromatic amino acid residues, eight of which are present on the surface of the inner sheet and provide a hydrophobic platform for interaction with neighboring subunits.

TRAIL, as the basis for developing therapeutic constructs, has several advantages over other apoptosis-inducing ligands. The main feature of TRAIL is the lack of cytotoxicity to normal cells, in contrast to a Fas ligand and TNF. Presumably, this is associated with the specificity of TRAIL to decoy the receptors DcR1 and DcR2 located on the surface of normal cells [32]. They inhibit apoptosis by competing with DR4 and DR5 for binding to TRAIL. Also, the DcR2 receptor can bind to DR4 to form a ligand-independent complex [33]. However, it remains unclear what else ensures the survival of normal cells, since decoy receptors are also found on tumor cells sensitive to TRAIL.

## TUMOR CELL RESISTANCE TO TRAIL

There are various causes for the resistance to TRAIL. Many molecules that regulate the apoptotic signal generation can act as its inhibitors. These molecules include the FLIP protein, inhibitors of apoptosis proteins (IAPs), the transcription factor NF-kB, etc. [34].

Overexpression of anti-apoptotic proteins belonging to the Bcl-2 family may contribute to the development of resistance to TRAIL in various tumor cells [35]. Association of cleaved c-FLIP with the DISC was found to prevent activation of caspase 8 [36]. TRAIL resistance may also be caused by various mutations in the proteins involved in the apoptosis signaling pathway: For example, mutations in the pro-apoptotic protein Bax lead to the resistance displayed by colon cancer epithelial cells [37].

For example, TRAIL-sensitive neuroectodermal tumor (PNET) cell lines express the necessary amounts of mRNA and caspase 8, while TRAIL-resistant PNET cells do not express them, which is a result of the methylation of the gene encoding caspase. It was noted that TRAIL-resistant PNET cells preserve their resistance even upon overexpression of TRAIL receptors [38, 39].

A high level of the transcription factor NF-kB in tumor cells may induce not only an increased expression of DR4 and DR5 receptors [40], but also the development of resistance to TRAIL, which is caused by increased synthesis of the anti-apoptotic proteins regulated by the factor [41].

The described variants do not encompass all the ways in which tumor cells develop resistance. Overcoming this resistance is the main thrust in the development of new agents that can activate DR4 and DR5 receptors.

## TRAIL-R AGONISTS IN CANCER THERAPY

To date, a variety of strategies targeting TRAIL-R have been developed. These include various forms of recombinant soluble human TRAIL (Apo2L or AMG- 951/dulanermin), DR4 and DR5 agonist antibodies, etc. [42]. These agents are safe and well tolerated by patients [43, 44].

An ideal therapeutic agent to activate TRAIL-dependent apoptosis should have activity comparable to that of the natural ligand, high antibody-like affinity to the receptor, and an elimination half-life sufficient to circulate in the bloodstream for a long time. Recombinant human TRAIL activates both death receptors, but its use is limited by its rapid hydrolysis in blood and short elimination half-life. In addition, TRAIL can bind to decoy receptors that are able to inhibit the activation of apoptosis [45]. As an alternative to TRAIL, antibodies capable of interacting only with death receptors and that do not affect decoy receptors have been developed. They are relatively safe, have improved pharmacokinetic properties compared to those of recombinant TRAIL, but they are specific only to one type of receptors. Despite the existing limitations, a variety of agents affecting death receptors, both as monotherapy and combination therapy, are now undergoing clinical trials.

The first recombinant version of TRAIL contained a TNF homologous domain with a polyhistidine tag [46] or a FLAG epitope [47] attached to the N-terminus. These fragments improve the protein purification process. Although these two modified proteins have demonstrated efficacy both in *in vitro* and *in vivo* trials, their use is hampered by their toxicity to liver hepatocytes.

To increase the stability of the TRAIL complex, several modifications have been developed. One of the approaches is to connect TRAIL with a leucine zipper motif (LZ-TRAIL) or an isoleucine zipper motif (iz- TRAIL). A similar approach is to link TRAIL with tenascin-C for the stabilization and oligomerization of the molecule. These agents have exhibited greater *in vivo *and *in vitro *activity compared to that of dulanermin, and they did not affect hepatocytes [48].

More recently, several research groups have developed a new TRAIL stabilization principle based on single-chain TRAIL (scTRAIL) [49]. In this approach, a molecule is initially expressed as a trimer in which three domains are interlinked in a head-to-tail fashion. An initially correctly assembled construct excludes the possibility of errors during its expression and prevents non-specific interaction with other molecules. This provides advantages to scTRAIL over its analogues and demonstrates efficacy against certain drug-resistant tumor lines.

Another approach for increasing the elimination half-life of TRAIL is to link TRAIL with molecules that have better pharmacokinetic properties, e.g. human serum albumin (HSA) or polyethylene glycol (PEG). According to the results of *in vivo *studies, pegylation of iz-TRAIL increases the elimination half-life, stability, and solubility of the molecule [50].

## ANTIBODIES

Antibodies to TRAIL-R1 (mapatumumab [51]) and TRAIL-R2 (conatumumab [52], lexatumumab [53], tigatuzumab [54], and drozitumab [55]) have demonstrated a degree of efficacy in preclinical trials. In clinical trials, all the antibodies exhibited safety and greater stability compared to those of TRAIL. Antibodies that had been effective in phase I clinical trials were studied in phase II clinical trials both as monotherapy and as combination chemotherapy with cisplatin, paclitaxel [56], and other anticancer agents.

The antibodies mapatumumab and conatumumab proved effective as monotherapy. In mapatumumab antibody therapy, clinical improvement was observed in 14 of 17 patients with non-Hodgkin lymphoma. Prolonged remission was observed in 29% of patients with non-small cell lung cancer and in 32% of patients with colorectal cancer [57, 58].

The combination of conatumumab with paclitaxel and carboplatin as first line treatment for patients with non-small cell lung cancer was more effective compared to a treatment with carboplatin and paclitaxel alone [59]. By contrast, mapatumumab, combined with paclitaxel and carboplatin, did not increase the efficacy of the treatment [60].

Furthermore, conatumumab was effective in combination with standard FOLFIRI chemotherapy and ganitumab as second line treatment for colorectal cancer, increasing the survival rate in patients in remission [61].

Tigatuzumab (CS-1008), combined with gentamicin, was well tolerated in the treatment of metastatic liver cancer, and the overall percentage of patients with an objective response rate amounted to 13.1% [62].

A recombinant analogue of the death receptor ligand dulanermin was tested in patients with different tumors and demonstrated activity against chondroblastoma, colorectal cancer, etc., during pre-clinical trials. Unfortunately, no similar efficacy was detected in clinical trials [63].

According to the presented data, effective treatment of cancer with death receptor agonists requires an individualized approach to each patient, because there is a risk of tumor cell resistance to such therapy. One of the principles for overcoming the resistance may be to search for the specific biomarkers of resistance, which could help characterize cells with high expression levels of death receptors, which would be sensitive to antibodies [66].

One of such approaches is the use of genetically modified T cells. T cells expressing a chimeric antigen receptor (CAR) of a TRAIL receptor single-chain antibody were capable of specific elimination of tumor cells with DR4. During interaction with tumor cells, the CAR-modified T cells were shown to trigger not only a DR4-induced apoptotic pathway, but also the mechanisms of T cell cytotoxicity [64, 65].

## PEPTIDE AGONISTS OF DEATH RECEPTORS

A promising approach is the search for appropriate peptide agonists of DR4 and DR5. The advantage of peptides over TRAIL is their ability to bind only to a certain death receptor [67]. Peptide ligands are screened using a phage display technology that selects peptides with agonistic properties based on a linkage between a genotype and a phenotype. The produced peptides, in both monomeric and dimeric forms, can bind to a receptor and activate it.

By using phage display, a group of researchers selected a YCKVILTHRCY peptide that was able to bind specifically to DR5. Tyr residues were added to the ends of the peptide to increase its solubility. The peptide properties were investigated both in the monomeric and dimeric (two covalently bound monomers) forms. Both forms were demonstrated to interact with DR5 and induce apoptosis in tumor cells of the Colo205 line. The effectiveness of the monomer may be associated with the fact that the peptide contains numerous hydrophobic residues and, at high concentrations, may aggregate in an aqueous medium [68]. Another research group also used phage display to select a GRVCLTLCSRLT peptide with high affinity for DR5 (IC_50_ = 30 nM). A LTL amino acid sequence was found to play a key role in the interaction with the receptor [69].

## CONCLUSION

Currently, there exist many approaches for affecting tumor cells, in particular through apoptotic pathways. Unfortunately, many of these approaches remain inappropriate due to cell resistance, as well as the inefficiency and instability of therapeutic agents. Other agents offer new opportunities for the treatment of tumor diseases. A more detailed investigation of the complex mechanism involving death receptor signaling pathways will boost the development of new agents that could be capable of overcoming the resistance and selectively affect cancer cells. On the other hand, effective use of existing death receptor antibodies requires a more detailed investigation of their application in combination therapy.

**SUPPLEMENT:** Classification of the DR agonists being tested in clinical trials

Disease	Phase/Adjunct therapy	Patients	Clinical efficacy	Reference
Lexatumumab fully IgG1-kappa human monoclonal agonistic antibody directed against DR5				
Solid cancer tumors	I	24	Low	[53]
	I	32	Low	[70]
	Ib/gemcitabine, pemetrexed, doxorubicin, or FOLFIRI	41	No data	
Mapatumumab fully IgG1-kappa human monoclonal agonistic antibody directed against DR4				
Solid cancer tumors	I	49	Yes	
	I	41	No	[71]
	I/paclitaxel, carboplatin	27	No	[72]
NHL	Ib/II	40	Low	[57]
CRC	II	38	Low	[58]
Cervical cancer	Ib/II/cisplatin, gemcitabine	49	Yes	
NSCLC	II	32	Low	
	II/paclitaxel, carboplatin	100	No	[60]
HCC	II/sorafenib	101	In progress	[73]
MM	II/bortezomib (velcade)	105	No data	
Conatumumab (AMG 655) fully IgG1-kappa human monoclonal agonistic antibody directed against DR5				
Solid cancer tumors	I	37	Yes	[74]
	I/increased dose	18		[47]
	Ib/AMG 479 (IGF-IR antagonist)	108		
NSCLC	II/paclitaxel, carboplatin	150	No	[59]
Lymphoma	II/bortezomib, vorinostat	20	No	
Soft tissue sarcoma	I/doxorubicin	6		[75]
	II/doxorubicin	120	Low	
	II/FOLFOX6, bevacizumab, ganitumab		In progress	
CRC	II/FOLFIRI, ganitumab	155	Yes	[61]
	II/mFOLFOX, bevacizumab	180	No	[76]
	I, II/panitumumab	53	No	
Pancreatic cancer	II/gemcitabine	125	Low	[77]
Tigatuzumab (CS-1008) fully IgG1-kappa human monoclonal agonistic antibody directed against DR5				
Carcinoma	I	17	No	[78]
NSCLC	II/paclitaxel, carboplatin	97	No	[54]
Pancreatic cancer	II/gemcitabine	62	No	[62]
Breast cancer	II/paclitaxel	64	Low	[79]
HCC	II/sorafenib	163	No	[80]
CRC	I	19	Low	[81]
Metastatic breast cancer	II/abraxane		In progress	
Drozitumab fully IgG1-kappa human monoclonal agonistic antibody directed against DR5				
Metastatic colorectal cancer	I/mFOLFOX, bevacizumab		Low	[82]
Dulanermin (rhApo2L/TRAIL) pro-apoptotic receptor agonist				
Solid cancer tumors	I	58	Low	[63]
NSCLC	Ib/paclitaxel, carboplatin+bevacizumab	24		[83]
	II/paclitaxel, carboplatin+bevacizumab	213	No	[84]
CRC	Ib/FOLFOX6+bevacizumab	23	Yes	[85]
	II/FOLFOX6+bevacizumab		In progress	
NHL	Ib/rituximab	7	Yes	[86]
	II/rituximab	132	In progress	
PRO95780 fully IgG1-kappa human monoclonal agonistic antibody directed against DR5				
Solid cancer tumors	I	50	No	[87]
CRC	I/FOLFOX, bevacizumab	6		
NHL	I/rituximab	49	No data	
CRC	I/bevacizumab, cetuximab, FOLFIRI, irinotecan	23	No data	
Chondrosarcoma	II		In progress	
NSCLC	II/paclitaxel, carboplatin, bevacizumab	128	No data	
NSCLC – non-small cell lung cancer; NHL – non-Hodgkin lymphoma; CRC – colorectal cancer; HCC – hepatocellular carcinoma; MM – multiple myeloma				

^#^The structure was solved by NMR in contrast to the other structures solved by X-ray crystallography.

## Abbreviations

DR4: death receptor 4
DR5: death receptor 5
TNF: tumor necrosis factor
